# Graded Elevation of c-Jun in Schwann Cells *In Vivo*: Gene Dosage Determines Effects on Development, Remyelination, Tumorigenesis, and Hypomyelination

**DOI:** 10.1523/JNEUROSCI.0986-17.2017

**Published:** 2017-12-13

**Authors:** Shaline V. Fazal, Jose A. Gomez-Sanchez, Laura J. Wagstaff, Nicolo Musner, Georg Otto, Martin Janz, Rhona Mirsky, Kristján R. Jessen

**Affiliations:** ^1^Department of Cell and Developmental Biology, University College London, London WC1E 6BT, United Kingdom,; ^2^Enzo Life Sciences, 4415 Lausen, Switzerland,; ^3^University College London Great Ormond Street Institute of Child Health, London WC1N1EH, United Kingdom, and; ^4^Max Delbrück Center for Molecular Medicine and Charité, University Hospital Berlin, Campus Benjamin Franklin, 13092 Berlin, Germany

**Keywords:** c-Jun, myelin, PNS, regeneration, Schwann, tumorigenesis

## Abstract

Schwann cell c-Jun is implicated in adaptive and maladaptive functions in peripheral nerves. In injured nerves, this transcription factor promotes the repair Schwann cell phenotype and regeneration and promotes Schwann-cell-mediated neurotrophic support in models of peripheral neuropathies. However, c-Jun is associated with tumor formation in some systems, potentially suppresses myelin genes, and has been implicated in demyelinating neuropathies. To clarify these issues and to determine how c-Jun levels determine its function, we have generated c-Jun OE/+ and c-Jun OE/OE mice with graded expression of c-Jun in Schwann cells and examined these lines during development, in adulthood, and after injury using RNA sequencing analysis, quantitative electron microscopic morphometry, Western blotting, and functional tests. Schwann cells are remarkably tolerant of elevated c-Jun because the nerves of c-Jun OE/+ mice, in which c-Jun is elevated ∼6-fold, are normal with the exception of modestly reduced myelin thickness. The stronger elevation of c-Jun in c-Jun OE/OE mice is, however, sufficient to induce significant hypomyelination pathology, implicating c-Jun as a potential player in demyelinating neuropathies. The tumor suppressor P19^ARF^ is strongly activated in the nerves of these mice and, even in aged c-Jun OE/OE mice, there is no evidence of tumors. This is consistent with the fact that tumors do not form in injured nerves, although they contain proliferating Schwann cells with strikingly elevated c-Jun. Furthermore, in crushed nerves of c-Jun OE/+ mice, where c-Jun levels are overexpressed sufficiently to accelerate axonal regeneration, myelination and function are restored after injury.

**SIGNIFICANCE STATEMENT** In injured and diseased nerves, the transcription factor c-Jun in Schwann cells is elevated and variously implicated in controlling beneficial or adverse functions, including trophic Schwann cell support for neurons, promotion of regeneration, tumorigenesis, and suppression of myelination. To analyze the functions of c-Jun, we have used transgenic mice with graded elevation of Schwann cell c-Jun. We show that high c-Jun elevation is a potential pathogenic mechanism because it inhibits myelination. Conversely, we did not find a link between c-Jun elevation and tumorigenesis. Modest c-Jun elevation, which is beneficial for regeneration, is well tolerated during Schwann cell development and in the adult and is compatible with restoration of myelination and nerve function after injury.

## Introduction

Schwann cell c-Jun has been implicated both in adaptive and maladaptive functions in peripheral nerves. In injured nerves, this transcription factor is a global amplifier of the repair Schwann cell phenotype and promotes regeneration and, in models of peripheral neuropathies, Schwann cell c-Jun supports axonal survival, trophic factor expression, and sensory–motor function (Arthur-Farraj et al., 2012, 2017; Hantke et al., 2014; Klein et al., 2014; Jessen and Mirsky, 2016). Reduced c-Jun levels in Schwann cells are also implicated in failure of regeneration due to aging and long-term denervation (Painter et al., 2014; Jessen and Mirsky, 2016). The c-Jun pathway is therefore of interest for the development of a pharmacology for nerve repair. Conversely, c-Jun is associated with tumor formation in some systems and potentially suppresses myelin genes (Eferl and Wagner, 2003; Parkinson et al., 2008). Based on this, it has been characterized as a negative regulator of myelination and implicated in demyelinating neuropathies (Jessen and Mirsky, 2008). In the present work, we have generated and analyzed mouse lines with graded expression of c-Jun in Schwann cells to clarify these issues and to determine how c-Jun levels determine its function.

c-Jun is present at low levels in Schwann cells of uninjured nerves, but is rapidly elevated 80- to 100-fold after nerve cut (De Felipe and Hunt, 1994; Shy et al., 1996; Parkinson et al., 2008; J. Gomez-Sanchez, K.R. Jessen, and R. Mirsky, unpublished data). c-Jun elevation is also seen in human neuropathies (Hutton et al., 2011). Although c-Jun is implicated in the promotion of a number of tumors, in other situations, it may have a role in the prevention of tumorigenesis by mechanisms that include activation of tumor suppressors such as P14^ARF^/p19^ARF^and Dmp1 (Eferl and Wagner, 2003; Ameyar-Zazoua et al., 2005; Shaulian, 2010). P19^ARF^ is elevated in Schwann cells after nerve transection and the striking activation of Schwann cell c-Jun after injury is not associated with tumor formation (Gomez-Sanchez et al., 2013; Jessen et al., 2015b). Rather, the role of c-Jun is to take part in controlling the conversion of myelin and Remak cells to Schwann cell specialized to perform injury-specific tasks and to promote repair (Jessen et al., 2015a; Jessen and Mirsky, 2016; Arthur-Farraj et al., 2017; Gomez-Sanchez et al., 2017). This includes preventing the death of injured neurons and promoting axon growth by expression of trophic factors, guiding axons back to their targets by forming regeneration tracks (Bungner bands), and breakdown of myelin directly by autophagy and indirectly by cytokine expression to recruit macrophages. Inactivation of Schwann cell c-Jun results in defective repair Schwann cells and impaired regeneration (Arthur-Farraj et al., 2012; Jessen and Mirsky, 2016).

The injury-induced extinction of myelin genes is also delayed without c-Jun, indicating that c-Jun has a dual function, promoting the expression of the repair phenotype and the suppression of the myelin phenotype. c-Jun suppression of myelin genes has only been studied directly in culture, where c-Jun suppresses the Krox20- or cAMP-induced activation of myelin genes, and enforced c-Jun inhibits myelination in cocultures (Parkinson et al., 2004, 2008). Negative transcriptional regulation of myelination has also been shown for Notch1 and Sox2 *in vivo* and suggested for other factors including Pax-3, Id2, and Sox-2 based on cell culture experiments (Jessen and Mirsky, 2008; Roberts et al., 2017).

The present results show that the function of c-Jun in Schwann cells depends on gene dosage, and that Schwann cells are surprisingly tolerant of the moderately (∼6-fold) elevated c-Jun seen in c-Jun OE/+ mice. In these mice, overexpression of c-Jun is sufficient to accelerate axonal regeneration (Wagstaff et al., 2017), so myelination and function are restored after nerve injury. Further, even high expression of c-Jun is not associated with tumor formation in Schwann cells, although this is sufficient to cause hypomyelination neuropathy.

## Materials and Methods

### 

#### Transgenic mice

Animal experiments conformed to UK Home Office guidelines under the supervision of University College London (UCL) Biological Services. To generate mice that overexpress c-Jun selectively in Schwann cells, female *R26c-Junstopf* mice, generated in the laboratory of Klaus Rajewsky, which carry a lox-P flanked STOP cassette in front of a CAG promoter-driven c-Jun cDNA in the ROSA26 locus, were crossed with male *P0Cre*^+/−^ mice (Feltri et al., 1999). This generated *P0-Cre*^+/−^*;R26c-Junstopf*^f/+^ mice, which we refer to as c-Jun OE/+ mice. These male mice were back-crossed with female *R26c-Junstopf*^f/f^ mice to generate *P0-Cre*^+^*;R26c-Junstopf*^f/f^ mice, referred to as c-Jun OE/OE mice. *P0-Cre*^−/−^ littermates were used as controls. Mice of either sex and on the C57BL/6 background were used in the experiments.

#### Genotyping

DNA for genotyping was extracted from ear or tail samples using the HotSHot method (Truett et al., 2000). Primers for genotyping the R26c-Junstopf transgene were 5′-TGGCACAGCTTAAGCAGAAA-3′ and 5′-GCAATATGGTGGAAAATAAC-3′ (270bp). The primers for the Rosa26 wild-type locus were 5′GGAGTGTTGCAATACCTTTCTGGGAGTTC-3′ and 5′TGTCCCTCCAATTTTACACCTGTTCAATTC-3′ (217bp band). The primers for the P0-Cre transgene were 5′-GCTGGCCCAAATGTTGCTGG-3′ and 5′CCACCACCTCTCCATTGCAC-3′ (480 bp band).

#### Nerve injury

The sciatic nerve was exposed and crushed (3 × 15 s at 3 rotation angles) at the sciatic notch using angled forceps. The wound was closed using veterinary autoclips. The nerve distal to the crush was excised for analysis at various time points. Contralateral uninjured sciatic nerves were used as controls for Western blotting, immunofluorescence, or electron microscopy.

#### Schwann cell culture

Schwann cell cultures were prepared from sciatic nerves of postnatal day 8 (P8)–P10 mouse pups essentially as in Morgan et al. (1991) and Arthur-Farraj et al. (2011). After enzymatic dissociation and centrifugation, the cell pellet was resuspended in defined medium (DM) (Meier et al., 1999) containing 10^−6^ m insulin and 5% horse serum (HS) and plated in drops on coverslips coated with poly-l-lysine and laminin. Cells were incubated at 37°C/5% CO_2_ and allowed to adhere for 24 h. After 24 h, the medium was changed to DM/0.5% HS (controls), DM with 10 ng/ml NRG1 alone, or DM with NRG1 10 ng/ml and dbcAMP 1 mm for 48 h before fixation and immunolabeling.

#### Antibodies

The following antibodies were used for Immunofluorescence: c-Jun (Cell Signaling Technology, rabbit 1:800, 9165, RRID: AB_2130165), Ki67 (Abcam, rabbit 1:100, ab15580, RRID: AB_443209), Krox20 (Covance, rabbit 1:100, PRB-236P, RRID: AB_10064079), SOX-10 (R&D Systems, goat 1:100, AF2864, RRID: AB_442208), donkey anti-goat IgG (H+L) Alexa Fluor 488 conjugate (Invitrogen, 1:1000, A11057, RRID: AB_2534104), Cy3 donkey anti-rabbit IgG (H+L) (Jackson Immunoresearch, 1:500, 711-165-152, RRID: AB_2307443), biotinylated anti-rabbit IgG (GE Healthcare, 1:600, RPN1004, RRID: AB_1062582), and Cy3 Streptavidin (Jackson ImunnoResearch, 1:500, 016-160-084).

The following antibodies were used for Western blot: GAPDH (Sigma-Aldrich, rabbit 1:5000, G9545, RRID: AB_796208), calnexin (Enzo Life Sciences, rabbit 1:1000, ADI-SPA-860-D, RRID: AB_2038898), c-Jun (Cell Signaling Technology, rabbit 1:1000, 9165), Krox20 (Millipore, rabbit 1:500, ABE1374, RRID: AB_2715555), Mpz (Aves Labs, chick 1:2000, PZO, RRID: AB_2313561), cyclin D1 (Santa Cruz Biotechnology, rabbit 1:200, sc-450, RRID: AB_627342), p19 Arf (5-c3-1) (Santa Cruz Biotechnology, rat 1:100, sc-32748, RRID: AB_628071), anti-mouse IgG HRP-linked (Promega, 1:2000, W4028, RRID: AB_430834), anti-rabbit IgG, HRP-linked (Cell Signaling Technology, 1:2000, 7074, RRID: AB_2099233), anti-rat IgG, HRP-linked (Cell Signaling Technology, 1:2000, 7077), and anti-chicken IgY, HRP-linked (Promega, 1:2000, G1351, RRID: AB_430845).

#### Immunohistochemistry

Schwann cells were fixed in 4% paraformaldehyde/PBS for 15 min and then immunolabeled. Transverse sciatic nerve cryosections (10 μm) were postfixed with 4% paraformaldehyde/PBS for 15 min, blocked in 0.2% Triton X-100 with 10% HS in PBS and subsequently incubated with primary antibodies in blocking solution overnight at 4°C, followed by 2 h in secondary antibodies and DAPI to identify cell nuclei (Thermo Fisher Scientific, 1:50,000). For Ki67 staining, biotin antibodies followed by Cy3 streptavidin were used.

#### Western blotting

For blotting, homogenates were obtained from injured and uninjured nerves and from cultured nerve segments essentially as described previously (Gomez-Sanchez et al., 2015). Experiments were repeated at least three times with fresh samples and representative photographs are shown. Densitometric quantification was by Image Lab 4.1 (Bio-Rad Laboratories). Measurements were normalized to loading control GAPDH and/or calnexin.

#### RNA sequencing analysis

##### Library preparation.

Total RNA was isolated using the RNeasy Lipid Tissue Mini Kit (Qiagen) with a DNase I step performed to eliminate traces of genomic DNA.The purified mRNA was fragmented and primed with random hexamers. Strand-specific first-strand cDNA was generated using reverse transcriptase in the presence of actinomycin D. The second cDNA strand was synthesized using dUTP in place of dTTP to mark the second strand. The resultant cDNA was then “A-tailed” at the 3′ end to prevent self-ligation and adapter dimerization. Truncated adaptors containing a T overhang were ligated to the A-tailed cDNA. Successfully ligated cDNA molecules were then enriched with limited cycle PCR (10–14 cycles). The high-fidelity polymerase used in the PCR is unable to extend through uracil. This means that only the first strand is amplified for sequencing, thus making the library strand specific.

##### Sequencing.

Libraries to be multiplexed in the same run were pooled in equimolar quantities. Samples were sequenced on the NextSeq 500 instrument (Illumina), resulting in ∼16 million reads per sample.

##### Data analysis.

Run data were demultiplexed and converted to fastq files using Illumina's bcl2fastq Conversion Software version 2.18 on BaseSpace. Fastq files were aligned to a reference genome using STAR on the BaseSpace RNA-Seq alignment app version 1.1.0. Reads per transcript were counted using HTSeq and differential expression was estimated using the BioConductor package DESeq2 (BaseSpace app version 1.0.0).

RNA Sequencing analysis was performed by UCL Genomics, UCL Great Ormond Street Institute of Child Health.

#### Electron microscopy

Nerves were processed as described previously (Gomez-Sanchez et al., 2015). Transverse ultrathin sections from neonatal, P7, and P21 sciatic nerve or adult (P60) or aged (P300) sciatic nerves or from injured distal stumps of adult sciatic nerves were taken 5 mm from the sciatic notch or cut site and mounted on film.

Photographs were taken using a Jeol 1010 electron microscope with a Gatan camera and software. Images were analyzed using ImageJ. Photographs at were taken at 3000× to measure the number of myelinated axons, non-myelinated axons bigger than 1.5 μm, and Schwann cell nuclei. The nerve area was measured from photographs taken at 200× magnification. Higher magnifications were used to show the Remak bundles or any abnormal morphology found in the nerve.

The percentage of extracellular matrix was analyzed using ImageJ software by converting electron microscopy images at 5000× and 10,000× into 8-bit images and tracing the presence of extracellular matrix.

#### Behavioral tests

Experiments conformed to UK Home Office guidelines. Nine or more mice/genotype were tested. Six-week-old mice were tested before surgery to ensure that there were no differences in normal responses between the genetic backgrounds. Sciatic function index (Inserra et al., 1998), toe spread reflex (Ma et al., 2011), and the toe pinch test, modified from Collier et al. (1961), were performed as in Arthur-Farraj et al. (2012).

#### Statistical analysis

Results are expressed as mean ± SEM. Statistical significance was estimated by one-way ANOVA with Tukey's correction, two-way ANOVA with Bonferroni's's multiple comparisons, Mann–Whitney *U* test, or Student's *t* test. *p* < 0.05 was considered statistically significant. Statistical analysis was performed using GraphPad Prism software (version 6.0).

## Results

### Adult uninjured nerves of c-Jun OE/+ and c-Jun OE/OE mice have high levels of c-Jun protein in Schwann cell nuclei

**Figure 1.**
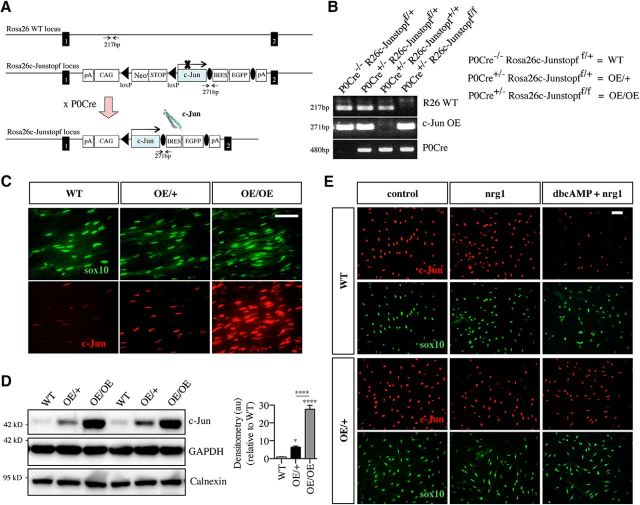
Graded overexpression of c-Jun Schwann cell nuclei of c-Jun OE/+ and c-Jun OE/OE mice. ***A***, Genomic structure of the c-Jun floxed allele in the Rosa26 locus. Excision of the stop codon is effected by crossing Rosa26c-Junff/+ mice with P0 cre-expressing mice to generate c-Jun OE/+ and c-Jun OE/OE mice overexpressing c-Jun specifically in Schwann cells. ***B***, PCR analysis showing the presence of c-Jun OE, Rosa26 WT, and P0 cre bands from DNA samples extracted from tails of WT, c-Jun OE/+, and c-Jun OE/OE mice. ***C***, Representative immunofluorescence images from WT, OE/+, and OE/OE sciatic nerve cryosections showing Sox10- and c-Jun-positive nuclei. Note the graded increase in c-Jun in c-JunOE/+ and c-Jun OE/OE mice. Scale bar, 50 μm. ***D***, Western blot of sciatic nerve protein extracts from P60 WT, c-Jun OE/+, and c-Jun OE/OE mice showing increasing c-Jun levels. The graph quantifies c-Jun expression in WT (*n* = 7), c-Jun OE/+ (*n* = 6), and c-Jun OE/OE (*n* = 6) mice. The quantifications are normalized to the levels in uninjured WT nerves, which are set as 1. Note that the difference in c-Jun expression between c-Jun OE/+ and c-Jun OE/OE nerves is also significant. One-way ANOVA with Tukey's comparison; *p < 0.05, ****p < 0.0001. ***E***, Representative immunofluorescence images from purified Schwann cell cultures from WT and c-Jun OE/+ mice. The cells were exposed to neuregulin (nrg) alone or neuregulin plus cAMP analog (dbcAMP), a combination that mimics axonal myelination signals. Note that neuregulin plus dbcAMP suppresses c-Jun in WT, but not in c-Jun OE/+, cells. Sox10 was used as a Schwann cell marker to show levels of c-Jun specifically in Schwann cells.

A diagrammatic representation of how the c-Jun-overexpressing mice were bred and produced is shown in Figure 1*A*. The *R26c-Junstopf* mouse has a c-Jun cDNA insert in the Rosa26 WT locus with two flanking loxP sites on either side of a STOP codon. These mice were bred with *P0Cre*^+/−^ mice (Feltri et al., 1999). In the presence of Cre recombinase, the STOP codon is removed and c-Jun is overexpressed specifically in Schwann cells (Feltri et al., 1999). *P0Cre*^−/−^*;R26c-Junstopf*^f/+^ control mice will be referred to as WT mice, *P0Cre*^+/−^*; R26c-Junstopf*^f/+^ will be referred to as c-Jun OE/+ mice, and *P0Cre*^+/−^*; R26c-Junstopf*^f/f^ will be referred to as c-Jun OE/OE mice. Genotyping of WT, c-Jun OE/+, and c-Jun OE/OE mice is shown in Figure 1*B*.

We examined c-Jun protein expression in adult (P60) uninjured sciatic nerves of c-Jun OE/+ and c-Jun OE/OE mice and compared this with that seen in WT mice. Double immunolabeling with c-Jun antibodies and Sox10 antibodies to identify Schwann cell nuclei specifically showed that Schwann cells in c-Jun OE/+ nerves expressed clearly elevated nuclear c-Jun levels compared with that seen in WT nerves, which showed barely detectable c-Jun using this staining protocol (see Materials and Methods). In c-Jun OE/OE nerves, nuclear c-Jun levels were further increased (Fig. 1*C*). No increase in c-Jun was seen in Sox10-negative nuclei labeled with DAPI (data not shown), indicating that c-Jun overexpression in these mouse lines was Schwann cell specific, in agreement with previous observations (Feltri et al., 1999).

Western blotting showed that c-Jun protein levels in uninjured adult sciatic nerves were elevated ∼6-fold in c-Jun OE/+ mice and ∼28-fold in c-Jun OE/OE mice compared with WT (Fig. 1*D*). In c-Jun OE/+ mice, c-Jun mRNA levels were 4.5-fold higher than in WT nerves.

These data indicate that the axonal signals that normally suppress c-Jun during myelination *in vivo* fail to supress c-Jun expression from the c-Jun OE transgene, as expected (Jessen and Mirsky, 2008; Parkinson et al., 2008). We verified this by exposing purified Schwann cell cultures to signals that mimic axonal myelin signals in mice, namely the combined activation of cAMP and neuregulin pathways (Arthur-Farraj et al., 2011). In these experiments, a combination of 1 mm dbcAMP and 10 nm neuregulin failed to suppress nuclear c-Jun expression in c-Jun OE/+ cells, although downregulation of c-Jun protein was seen in WT cells (Fig. 1*E*).

The elevation of c-Jun specifically in Schwann cell nuclei in c-Jun OE/+ and c-Jun OE/OE mice allowed us to study *in vivo* the effects of a graded increase in c-Jun expression on Schwann cells in uninjured and injured nerves.

### Transcriptional profiling of uninjured nerves in WT, c-Jun OE/+, and c-Jun OE/OE mice

**Figure 2.**
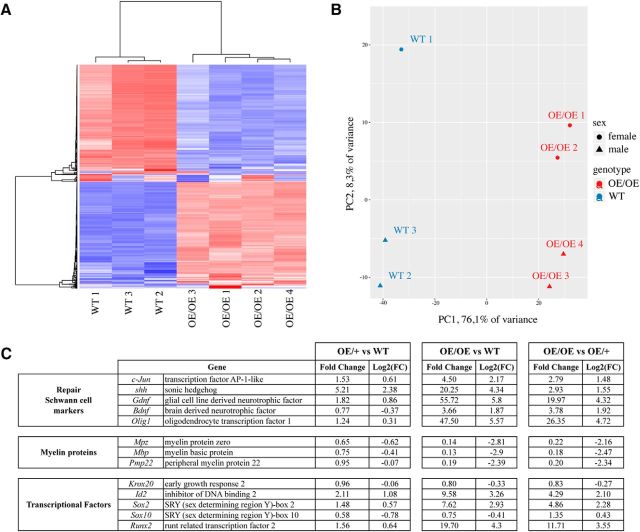
Gene expression in c-Jun OE/+ and c-Jun OE/OE mice. ***A***, Heat map of the 400 most regulated genes in uninjured nerves of WT and c-Jun OE/OE mice. ***B***, Principal component analysis map of gene regulation in WT and c-Jun OE/OE nerves. ***C***, Expression of 13 genes of interest in the sciatic nerve of OE/+ and OE/OE mice. The table shows how c-Jun elevation affects the expression of a subset of repair cell markers, myelin proteins, and transcription factors in the mouse lines indicated. Note that, in OE/+ nerves, only *Shh* is regulated ≥2-fold. In OE/OE nerves, repair cell markers are upregulated and myelin genes are downregulated, although two important myelin regulators, *Krox20* and *Sox10*, are not strongly affected. WT, *n* = 3; OE/+, *n* = 4; and OE/OE, *n* = 4.

**Figure 3.**
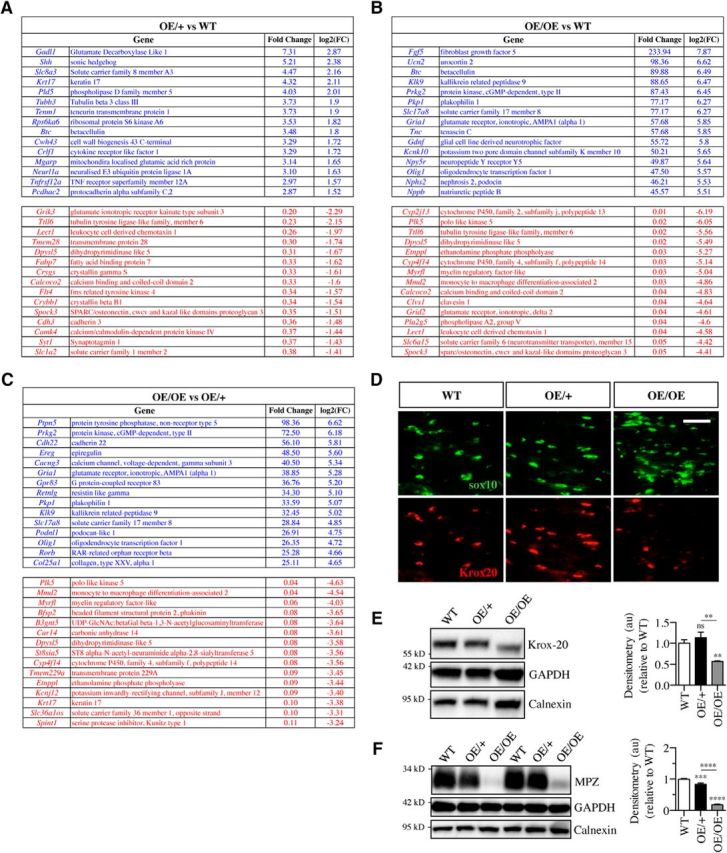
The 15 most upregulated and downregulated genes in the sciatic nerve of OE/+ and OE/OE mice. ***A***, ***B***, The 15 most strongly elevated genes (top) and the 15 most suppressed genes (bottom) in response to c-Jun elevation in the mouse lines indicated. ***C***, The 15 most strongly regulated gene in OE/OE nerves compared with expression in OE/+ nerves. ***D***, Representative immunofluorescence images from WT, c-Jun OE/+, and c-Jun OE/OE sciatic nerve cryosections showing Krox20 in Sox10-positive Schwann cell nuclei. Note similar Krox20 expression in WT and c-Jun OE/+ nerves, but much reduced levels in c-Jun OE/OE nerves. Scale bar, 50 μm. ***E***, Western blot of sciatic nerve protein extracts from P60 mice showing similar levels of Krox20 in WT and c-Jun OE/+ nerves, but lower levels in c-Jun OE/OE nerves. The graph quantifies Krox20 expression in WT (*n* = 5), c-Jun OE/+ (*n* = 4), and c-Jun OE/OE (*n* = 5) mice. The quantifications are normalized to the levels in uninjured WT nerves, which are set as 1. One-way ANOVA with Tukey's comparison: *p < 0.05, **p < 0.01. ***F***, Western blot of sciatic nerve protein extracts from P60 mice. Note that Mpz expression is 15% lower than WT in c-Jun OE/+ nerves, but strongly suppressed in c-Jun OE/OE nerves. The graph quantifies Mpz expression in WT (*n* = 5), c-Jun OE/+ (*n* = 4), and c-Jun OE/OE (*n* = 5) mice. The quantifications are normalized to the levels in uninjured WT nerves, which are set as 1. One-way ANOVA with Tukey's comparison: ****p < 0.0001.

To document changes in gene expression caused by c-Jun elevation in c-Jun OE/+ and OE/OE mice, we performed RNA sequencing analysis on uninjured adult (P60) sciatic nerves. Heat-map and principal component analysis confirmed that c-Jun overexpression was the dominant source of differential gene expression (Fig. 2*A*,*B*). In OE/+ nerves, which express ∼6-fold WT levels of c-Jun protein, 67 genes were ≥2-fold upregulated and 25 genes were ≥2-fold downregulated compared with WT nerves. Among 13 genes that we considered of particular interest, one gene, *Shh*, was upregulated ≥2-fold (Fig. 2*C*). *c-Jun* was expressed at 153% of WT levels and GDNF at 182% of WT levels and the myelin protein genes *Mbp* and *Mpz* were expressed at ∼65% and 75% of WT levels, respectively. The mRNA level for *Krox20 (Egr2)*, a key myelin regulator, was essentially unchanged. The 15 most upregulated and downregulated genes in c-Jun OE/+ are shown in Figure 3*A*.

In OE/OE nerves, which express ∼28-fold WT levels of c-Jun protein, 909 genes were ≥2-fold upregulated and 1055 genes were ≥2-fold downregulated compared with WT nerves. Most of the 13 genes of particular interest changed expression by ≥2-fold in these mice (Fig. 2*C*).This included *c-Jun*, which was elevated 4- to 5-fold; *Gdnf*, which was elevated by ∼56-fold; and *Shh* and *Olig1*, which were elevated 20-fold and 48-fold, respectively. The myelin protein genes *Mbp* and *Mpz* were reduced to 13–14% of WT levels. The 15 most upregulated and downregulated genes in c-Jun OE/OE nerves are shown in Figure 3*B*.

A comparison of gene expression between OE/+ and OE/OE mice with respect to the 13 genes of interest and the most regulated genes is shown in Figures 2*C* and 3*C*, respectively.

The fact that c-Jun protein was more strongly elevated in terms of fold change from WT than c-Jun mRNA in both c-Jun OE/+ and OE/OE mice suggests that posttranscriptional controls are important in controlling c-Jun levels. RNA-Seq data were deposited in ArrayExpress (https://www.ebi.ac.uk/arrayexpress/) with accession ID: E-MTAB-6138.

### Expression of myelin-related proteins in uninjured nerves of OE/+ and OE/OE mice

We examined two key myelin-related proteins, the promyelin transcription factor Krox20 (Egr2) and the myelin adhesion protein Pzero (Mpz), in uninjured sciatic nerves of c-Jun OE/− and OE/OE mice. Consistent with the mRNA data, Krox20 levels were essentially unaffected in c-Jun OE/+ mice both in double-label immunohistochemical experiments, which show Krox20 in Schwann cell nuclei, and in Western blotting experiments (Fig. 3*D*,*E*). Mpz levels in these mice were ∼15% lower than those found in WT mice (Fig. 3*F*). In contrast, the c-Jun OE/OE mice expressed significantly less Krox20 protein in Schwann cell nuclei and Western blots (Fig. 3*D*,*E*) and much reduced levels of Mpz (Fig. 3*F*).

This indicates that, in adult-Jun OE/+ nerves, the levels of key myelin-related proteins and their mRNA remain relatively mildly affected despite ∼6-fold elevation of Schwann cell c-Jun. This tolerance does, however, break down when c-Jun levels are elevated ∼28-fold, as seen in c-Jun OE/OE mice, which is consistent with the capacity of c-Jun to regulate myelin genes negatively as indicated in *in vitro* experiments and in mice with conditional inactivation of Schwann cell c-Jun (Parkinson et al., 2004, 2008; Arthur-Farraj et al., 2012).

### Structure of adult nerves is nearly normal in c-Jun OE/+ mice

Although the levels of myelin proteins were normal in c-Jun OE/+ mice, it remained possible that the substantial c-Jun elevation affected myelination and nerve architecture. This was tested by a morphometric comparison of WT and c-Jun OE/+ nerves.

**Figure 4.**
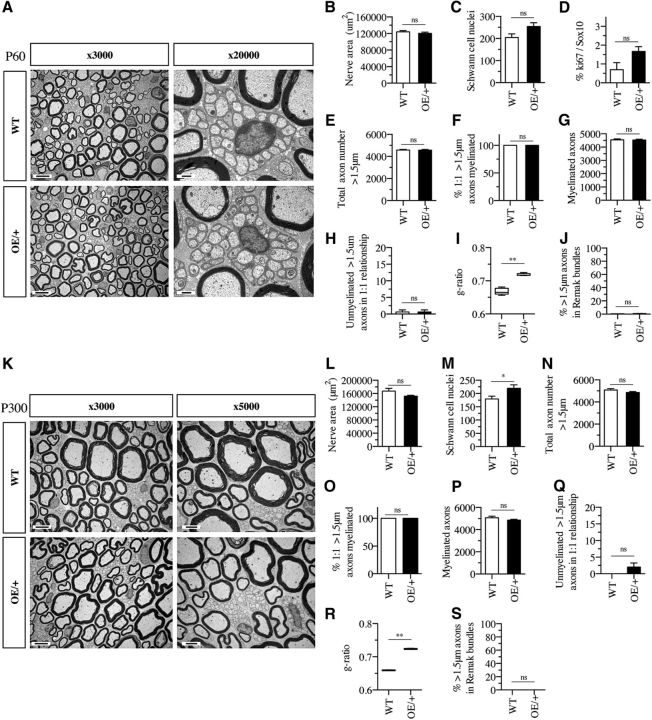
Electron microscopic structure of adult nerves in WT and OE/+ mice. ***A***, Electron micrographs showing similar overall appearance of nerves from P60 WT and OE/+ mice. Scale bar, 5 μm. ***B***, Total area of P60 WT and OE/+ mouse nerves is not significantly different. Mann–Whitney *U* test: *p* = 0.5317 (*n* = 5). ***C***, The number of Schwann cell nuclei per sciatic nerve profiles is not significantly different between P60 WT and OE/+ nerves. Mann–Whitney *U* test: *p* = 0.0952 (*n* = 5). ***D***, Counts of Ki67-positive/Sox10-positive nuclei indicate that the difference in Schwann cell proliferation between WT and c-Jun OE/+ mice is not significantly different. Mann–Whitney *U* test: *p* = 0.2000 (*n* = 3). ***E***, The total number of axons larger than 1.5 μm in diameter is similar in P60 WT and OE/+ nerves. Mann–Whitney *U* test: *p* = 0.9444 (*n* = 5). ***F***, The percentage of axons in a 1: 1 relationship and >1.5 μm in diameter that are myelinated is similar in P60 WT and OE/+ nerves. Mann–Whitney *U* test: *p* > 0.9999 (*n* = 5). ***G***, Per nerve profile, the number of myelinated axons is similar in P60 WT and OE/+ nerves. Mann–Whitney *U* test: *p* = 0.8016 (*n* = 5). ***H***, Per nerve profile, the number of axons in a 1:1 relationship and >1.5 μm in diameter but not myelinated is not significantly different between P60 WT and OE/+nerves. Mann–Whitney *U* test: *p* > 0.999 (*n* = 5). ***I***, Myelin thickness measured by g-ratios is thinner in P60 OE/+ nerves compared with WT. The whiskers extend from the 5th to the 95th percentiles. Mann–Whitney *U* test: *p* = 0.0079 (*n* = 5). ***J***, The percentage of axons >1.5 μm in diameter that remain unmyelinated and within Remak bundles is very low and similar in P60 WT and OE/+ nerves. Mann–Whitney *U* test: *p* = 0.1508 (*n* = 5). ***K***, Electron micrographs showing that the overall structure of adult P300 nerves in WT and OE/+mice is similar. Scale bar, 5 μm. ***L***, The area of transverse profiles of P300 WT (*n* = 4) and OE/+ (*n* = 5) nerves is not statistically different. Mann–Whitney *U* test: *p* = 0.2857. ***M***, The number of Schwann cell nuclei per sciatic nerve profile is somewhat higher in P300 OE/+ (*n* = 5) nerves compared with WT (*n* = 4). Mann–Whitney *U* test: *p* = 0.0317. ***N***, The total number of axons larger than 1.5 μm in diameter is similar in P300 WT (*n* = 4) and c-Jun OE/+ (*n* = 5) nerves. Mann–Whitney *U* test: *p* = 0.1905. ***O***, The percentage of axons in a 1:1 relationship and >1.5 μm in diameter that are myelinated is similar in P300 WT (*n* = 4) and OE/+ (*n* = 5) nerves. Mann–Whitney *U* test: *p* = 0.4444. ***P***, The numbers of myelinated axons per nerve profile is similar in P300 WT (*n* = 4) and OE/+ (*n* = 5) nerves. Mann–Whitney *U* test: *p* = 0.1905. ***Q***, Per nerve profile, the number of axons that are >1.5 μm in diameter and in a 1:1 relationship but not myelinated is not significantly different between P300 WT (*n* = 4) and OE/+ (*n* = 5) nerves. Mann–Whitney *U* test: *p* = 0.4444. ***R***, Measured by g-ratios, myelin is thinner in P300 OE/+ (*n* = 5) nerves than in WT (*n* = 4). The whiskers extend from the 5th to the 95th percentiles. Mann–Whitney *U* test: *p* = 0.0079. ***S***, The percentage of unmyelinated axons >1.5 μm in diameter that remain in Remak bundles is similar in P300 WT (*n* = 4) and OE/+ (*n* = 5) nerves. Mann–Whitney *U* test: *p* > 0.9999.

The general appearance of WT and c-Jun OE/+ nerves of 60-d-old mice was similar (Fig. 4*A*). The size of the cross-sectional profiles of the sciatic nerve and the number of Schwann cell nuclei were not significantly different between the two genotypes and Ki67 labeling of Sox10-positive cells failed to show a significant increase in Schwann cell proliferation (Fig. 4*B–D*). WT and c-Jun OE/+ nerves had similar numbers of >1.5 μm axons per nerve profile and the percentage of segregated (1:1), myelin-competent (>1.5 μm diameter) axons that were myelinated and the total number of myelinated axons were comparable (Fig. 4*E–G*). Both WT and c-Jun OE/+ nerves contained similar, very low numbers of myelin-competent (>1.5 μm diameter) axons in a 1:1 relationship that remained unmyelinated (Fig. 4*H*). Measurements of g-ratios showed that myelin sheaths were slightly thinner in c-Jun OE/+ mice compared with WT (Fig. 4*I*). Remak bundles in c-Jun OE/+ mice appeared normal and the percentage of >1.5 μm axons that were found within Remak bundles was similar and very low in both genotypes (Fig. 4*J*).

We found that this similarity between WT and c-Jun-overexpressing c-Jun OE/+ 60-d-old mice remained even in old (300 d) mice (Fig. 4*K–S*). As in young mice, observations of general appearance and quantitative analysis failed to reveal significant differences between the two genotypes, except for the difference in G-ratios, a difference that was also seen in young mice (Fig. 4*R*). The only age-induced change related to Schwann cell numbers, which were somewhat elevated in old mice, with the difference between the genotypes reaching statistical significance (Fig. 4*M*).

These observations show that, although c-Jun OE/+ mice show ∼6-fold elevation of c-Jun protein that is localized to Schwann cell nuclei, they achieve essentially normal Schwann cell and nerve architecture, with the exception of modestly reduced myelin thickness.

### Adult nerves of c-Jun OE/OE mice are hypomyelinated and show onion bulbs and hyperplasia but do not form tumors

**Figure 5.**
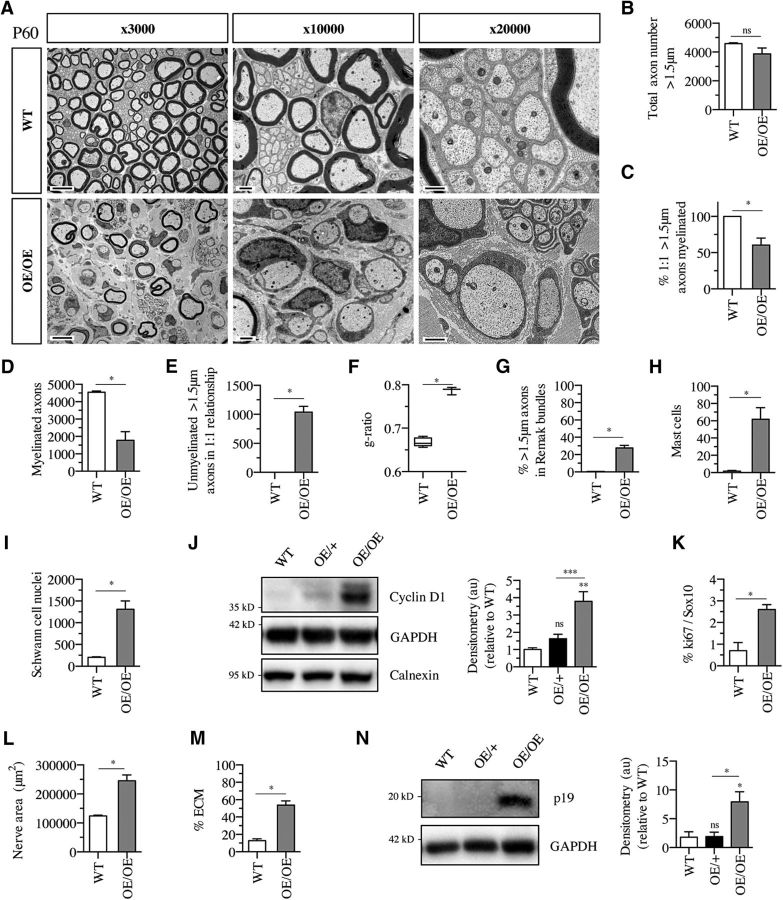
High c-Jun levels in c-Jun OE/OE nerves result in hypomyelination. ***A***, Electron micrographs showing lack of myelin and increased connective tissue spaces in P60 c-Jun OE/OE nerves compared with WT. ***B***, The total number of axons larger than 1.5 μm in diameter is not significantly different in P60 WT (*n* = 5) and c-Jun OE/OE (*n* = 3) nerves. Mann–Whitney *U* test: *p* = 0.0714. ***C***, The percentage of axons in a 1: 1 relationship and >1.5 μm in diameter that are myelinated is lower in c-Jun OE/OE mice than in WT (*n* = 5) and OE/OE (*n* = 3) nerves. Mann–Whitney *U* test: *p* = 0.0179. ***D***, The number of myelinated axons per nerve profile is substantially reduced in c-Jun OE/OE (*n* = 3) nerves compared with WT (*n* = 5). Mann–Whitney *U* test: *p* = 0.0357. ***E***, Per nerve profile, the number of axons in a 1:1 relationship and >1.5 μm in diameter but not myelinated is much higher in OE/OE (*n* = 3) nerves than in WT (*n* = 5). Mann–Whitney *U* test: *p* = 0.0179. ***F***, Myelin, measured as g-ratios, is thinner in OE/OE (*n* = 3) mice compared with WT (*n* = 5). The whiskers extend from the 5th to the 95th percentiles. Mann–Whitney *U* test: *p* = 0.0357. ***G***, The percentage of unmyelinated axons >1.5 μm in diameter that remain in Remak bundles is higher in OE/OE (*n* = 3) nerves than in WT (*n* = 5) nerves. Mann–Whitney *U* test: *p* = 0.0357. ***H***, Nerves in c-Jun OE/OE mice (*n* = 3) contain more mast cells that nerves in WT mice (*n* = 5). Mann–Whitney *U* test: *p* = 0.0179. ***I***, OE/OE (*n* = 3) nerves show more Schwann cell nuclei per nerve profile than WT (*n* = 5) nerves. Mann–Whitney *U* test: *p* = 0.0357. ***J***, Western blot of sciatic nerve protein extracts from P60 mice. Note that levels of cyclin D1 (a marker of cell proliferation) are significantly higher in OE/OE nerves than in WT or OE/+ or nerves. The results are quantified in the graph. WT, *n* = 5; OE/+, *n* = 4; and OE/OE, *n* = 5. The quantifications are normalized to the levels in uninjured WT nerves, which are set as 1. One-way ANOVA with Tukey's comparison: *p < 0.05, **p < 0.01. ***K***, Counts of Ki67-positive/Sox10-positive nuclei indicate a higher rate of Schwann cell proliferation in c-Jun OE/OE mice (*n* = 5) compared with WT (*n* = 3). Mann–Whitney *U* test: *p* = 0.0179. ***L***, The area of transverse profiles of OE/OE (*n* = 3) nerves is larger than of WT nerves. Mann–Whitney *U* test: *p* = 0.0357 (*n* = 5). ***M***, Tracing of cell profiles and extracellular space (ECM) in transverse nerve sections, followed by area measurements, shows a relative increase in extracellular space in OE/OE (*n* = 3) nerves compared with WT (*n* = 5). Mann–Whitney *U* test: *p* = 0.0357. ***N***, Western blot of sciatic nerve protein extracts from P60 mice. Note increased expression of the tumor suppressor p19ARF. The results are quantified in the graph. The quantifications are normalized to the levels in uninjured WT nerves, which are set as 1. One-way ANOVA with Tukey's comparison: *p < 0.05 (*n* = 3).

In contrast to c-Jun OE/+ mice, the higher (∼28-fold) c-Jun expression in c-Jun OE/OE mice resulted in obvious lack of myelin in 60-d-old mice (Fig. 5*A*). Although the total number >1.5 μm axons was similar to WT (Fig. 5*B*), the percentage of segregated (1:1), myelin-competent (>1.5 μm diameter) axons that were myelinated was reduced by ∼40% (Fig. 5*C*) and there was a corresponding increase in the number of myelin-competent axons that that had reached a 1:1 relationship but remained unmyelinated (Fig. 5*E*). The myelin sheaths in c-Jun OE/OE mice were also thin compared with WT (Fig. 5*F*). Although all of this indicates an impediment to myelination, a sorting defect was indicated by the fact that the percentage of >1.5 μm axons that remained within Remak bundles was strikingly increased to ∼28% compared with <1% in WT (Fig. 5*G*). As a result of impaired myelination and sorting, the number of myelinated axons in c-Jun OE/OE nerves was substantially lower than in WT nerves (Fig. 5*D*). As seen in mouse models of CMT1A neuropathy and a number of other mouse mutants with elevated, nontumorigenic Schwann cell proliferation, the organization of Remak bundles was somewhat altered (Robertson et al., 2002; Chen et al., 2003; Ling et al., 2005; Verhamme et al., 2011). The cells sometimes showed increased membranous structures and processes that were not in contact with axons and they contained fewer axons per transverse section of a bundle, suggesting the presence of a larger number of Remak cells each taking care of fewer axons (Fig. 5*A*). Increased mast cell numbers are seen in several neuropathic conditions and mutant models and after mechanical nerve injury (Olsson, 1971; Ling et al., 2005; Ishii et al., 2016). We therefore counted mast cell numbers and found substantial elevation in c-Jun OE/OE nerves (Fig. 5*H*).

An increase in Schwann cell number, a feature of many neuropathies including CMT1A (Robertson et al., 2002; Lupski and Chance, 2005), was also seen in c-Jun OE/OE nerves, the total number of Schwann cell nuclei per nerve profile being ∼6-fold that in WT (Fig. 5*I*). Western blots of Cyclin D1 indicated ongoing proliferation among the cells of c-Jun OE/OE nerves (Fig. 5*J*). Proliferation of Schwann cells was indicated in double immunolabeling with Ki67 and Sox10 antibodies to detect dividing Schwann cells because double labeled Schwann cells, although few, were ∼3× more common in c-Jun OE/OE nerves than in WT nerves (Fig. 5*K*). Observations in the electron microscope provided no evidence for the presence of a significant number of Schwann cells without contact with axons. The increased number of Schwann cell nuclei in nerve sections is likely due to non-myelinating cells in a 1:1 ratio, with axons being shorter than myelin cells, cells with thin myelin sheaths being shorter than those with normal sheath thickness, and an increased number of Remak cells.

The sciatic nerves of 60-d-old c-Jun OE/OE mice were enlarged, showing total cross-sectional profiles that were ∼2× that in c-Jun OE/+ or WT nerves (Fig. 5*L*). Collagen containing extracellular space was also markedly increased in c-Jun OE/OE nerves, occupying 133,349 μm^2^ (±19,891; *n* = 3; 54% of nerve area) in 60 d c-Jun OE/OE nerves, but only 16,069 μm^2^ (±2834; *n* = 5; 13% of nerve area) in WT nerves (Fig. 4*M*). This amounts to an increase in extracellular space of 116,280 μm^2^. Because the nerves of c-Jun OE/OE nerves are 121,486 μm^2^ larger than WT nerves, >95% of the enlargement seen in c-Jun OE/OE nerves is due to increased collagen-containing extracellular space, with a likely contribution from increased number of Remak cells and cells other than Schwann cells. Increase in endoneurial connective tissue is seen in a number of neuropathies, including CMT1A, and in the *trembler* and *twitcher* mouse mutants (Low, 1977; Palumbo et al., 2002; Ling et al., 2005; Kagitani-Shimono et al., 2008; Fledrich et al., 2012).

We examined the mutant nerves extensively for the presence of tumors or cellular arrangements reminiscent of tumor formation, but failed to find any evidence for either. Consistent with this, the tumor suppressor p19^ARF^ was strongly elevated in uninjured nerves of c-Jun OE/EO mice (Fig. 5*N*).

**Figure 6.**
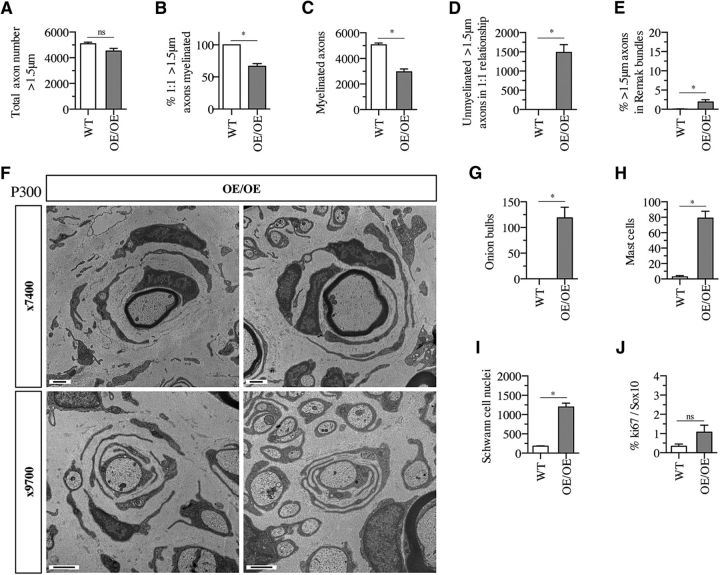
Nerves of aged c-Jun OE/OE mice. ***A***, The nerves of P300 c-Jun OE/OE mice and WT mice contain comparable numbers of axons. Mann–Whitney *U* test: *p* = 0.1143 (*n* = 4). ***B***, In P300 c-Jun OE/OE mice, the percentage of axons in a 1: 1 relationship and >1.5 μm in diameter that are myelinated is lower than in WT mice. Mann–Whitney *U* test: *p* = 0.0286 (*n* = 4). ***C***, In P300 c-Jun OE/OE mice, the number of myelinated axons per nerve profile is reduced compared with WT. Mann–Whitney *U* test: *p* = 0.0286 (*n* = 4). ***D***, Per nerve profile, P300 c-Jun OE/OE mice have a much larger number of unmyelinated axons that are >1.5 μm in diameter and in a 1:1 relationship and compared with WT mice. Mann–Whitney *U* test: *p* = 0.0286 (*n* = 4). ***E***, The percentage of unmyelinated axons >1.5 μm in diameter that are found within Remak bundles is higher in OE/OE nerves than in WT nerves. Mann–Whitney *U* test: *p* = 0.0286 (*n* = 4). ***F***, Electron micrographs from nerves of P300 c-Jun OE/OE mice showing examples of onion bulbs. The central axon, which is sometimes myelinated (top), is surrounded by relatively few layers of flattened Schwann cells, suggesting an early stage of bulb formation. Scale bar, 1 μm. ***G***, The number of onion bulbs in P300 OE/OE nerves is much higher than in WT nerves. Mann–Whitney *U* test: *p* = 0.0286 (*n* = 4). ***H***, P300 OE/OE nerves contain a higher number of mast cells than WT nerves. Mann–Whitney *U* test: *p* = 0.0286 (*n* = 4). ***I***, Nerves in P300 c-Jun OE/OE mice show more Schwann cell nuclei per nerve profile than nerves in WT mice. Mann–Whitney *U* test: *p* = 0.0286 (*n* = 4). ***J***, The rate of Schwann cell proliferation is not significantly higher in P300 OE/OE nerves than in WT nerves, as shown by the counts of Ki67-positive/Sox10-positive nuclei. Mann–Whitney *U* test: *p* = 0.1000 (*n* = 3).

Examination of old (P300) mice showed that only three of the parameters studied above changed obviously with age (Fig. 6*A–J*): (1) the appearance of significant numbers of onion bulbs (Fig. 6*F*,*G*); (2) a reduction in Schwann cell proliferation, which was no longer significantly elevated (Fig. 6*J*); and (3) a reduction in the percentage of >1.5 μm axons that remained within Remak bundles, from ∼28% at P60 (Fig. 5*G*) to <2% (Fig. 6*E*). Therefore, in nerves of c-Jun OE/OE mice, a large number of axons appear to segregate gradually from Remak bundles between P60 and P300. The proportion of these >1.5 μm axons that myelinate is similar to that of the >1.5 μm segregated axons in P60 nerves: ∼60% (Fig. 6*B*). No tumors were found in older mice (*n* = 18).

### Developmental myelination is delayed in c-Jun OE/+ mice but inhibited in c-Jun OE/OE mice

Although adult nerves of c-Jun OE/− mice are essentially normal, we tested whether the c-Jun elevation in these nerves caused a delay in myelination during development. We also determined whether the lack of myelin in the adult c-Jun OE/OE nerves was due to demyelination in the adult or inhibition of myelination during development.

**Figure 7.**
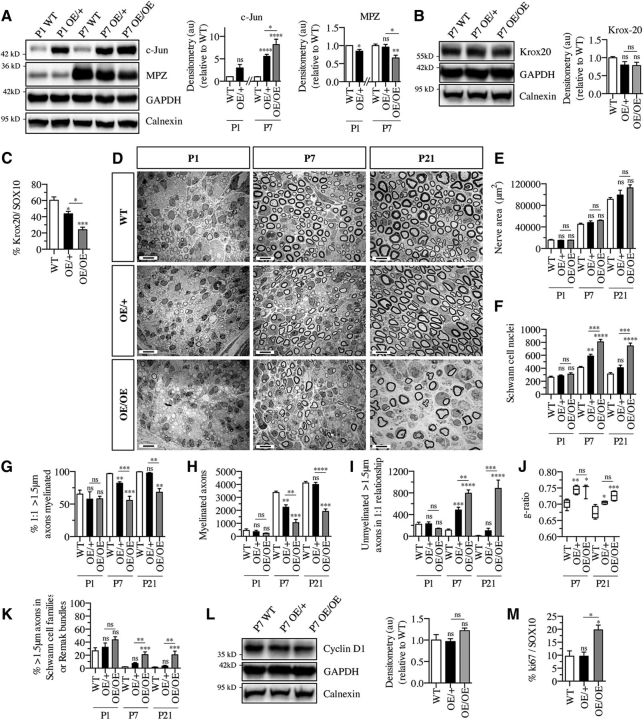
Developmental overexpression of c-Jun delays myelination in c-Jun OE/+ mice, but inhibits myelination in c-Jun OE/OE mice. ***A***, Western blot of nerve extracts from P1 and P7 sciatic nerves. The results are quantified in the graphs. Data from P1 nerves are normalized to levels in P1 WT nerve, which are set as 1, whereas data from P7 nerves are normalized to levels in P7 WT nerve, which are set as 1. Note that, by P7, c-Jun is elevated in both OE/+ and OE/OE nerves, whereas Mpz is reduced in OE/OE nerves only. P1 WT, *n* = 3; OE/+, *n* = 3; P7 WT, *n* = 4; OE/+, *n* = 4; and OE/OE, *n* = 3. Statistical analysis for P1 is Student's *t* test: *p* = 0.0608 for c-Jun, *p* = 0.0174 for Mpz. Statistical analysis for P7 is one-way ANOVA with Tukey's comparison: *p < 0.05, **p < 0.01, ****p < 0.0001. ***B***, Western blot of nerve extracts from P7 WT, OE/+, and OE/OE nerves. The results are quantified in the graph. Krox20 levels are similar in all genotypes. One-way ANOVA with Tukey's comparison: *p* = 0.2053 (*n* = 3). ***C***, The percentage of Krox20/Sox10-positive Schwann cells in sections from WT (*n* = 8), OE/+ (*n* = 6), and OE/OE (*n* = 3) sciatic nerves at P7. Note a graded decrease in Krox20-positive cells as levels of c-Jun increase. One-way ANOVA with Tukey's comparison: *p < 0.05, ***p < 0.001. ***D***, Representative electron micrographs from P1, P7, and P21 nerves of WT, c-Jun OE/+, and c-Jun OE/OE mice. Note hypomyelination in OE/OE nerve at P7 and P21 and transient hypomyelination in OE/+ nerves at P7. Scale bar, 5 μm. ***E***, The nerve areas are similar in all three genotypes at all developmental stages. One-way ANOVA with Tukey's comparison: P1 WT (*n* = 5), OE/+ (*n* = 4), and OE/OE (*n* = 5), *p* = 0.1978; P7 WT (*n* = 5), OE/+ (*n* = 5), and OE/OE (*n* = 3), *p* = 0.2261; and P21 WT (*n* = 5), OE/+ (*n* = 4), and OE/OE (*n* = 4), *p* = 0.084. ***F***, The number of Schwann cell nuclei per sciatic nerve profile at P1, P7, and P21 in nerves of WT, c-Jun OE/+, and c-Jun OE/OE mice. Note the transient difference between WT and OE/+ nerves at P7, whereas OE/OE nerves have more Schwann cells at P7 and P21. P1 WT, *n* = 5; OE/+, *n* = 4; and OE/OE, *n* = 5; P7 WT, *n* = 5; OE/+, *n* = 5; and OE/OE, *n* = 3; and P21 WT, *n* = 5; OE/+, *n* = 4; and OE/OE, *n* = 4. One-way ANOVA with Tukey's comparison: **p < 0.01, ***p < 0.001, ****p < 0.0001. ***G***, The percentage of axons in a 1:1 relationship and >1.5 μm in diameter that are myelinated at P1, P7, and P21 in nerves of WT, c-Jun OE/+, and c-Jun OE/OE mice. Note reduced myelination in OE/OE mice at P7 and P21 and transient reduction in OE/+ mice at P7. P1 WT, *n* = 5; OE/+, *n* = 4; and OE/OE, *n* = 5; P7 WT, *n* = 5; OE/+, *n* = 5; and OE/OE, *n* = 3; and P21 WT, *n* = 5; OE/+, *n* = 4; and OE/OE, *n* = 4;. One-way ANOVA with Tukey's comparison: **p < 0.01 and ***p < 0.001. ***H***, The number of myelinated axons per nerve profile at P1, P7, and P21 in nerves of WT, c-Jun OE/+, and c-Jun OE/OE mice. Note the substantial reduction in myelinated axons in OE/OE nerves at P7 and P21 and transient decrease in OE/+ mice at P7. P1 WT, *n* = 5; OE/+, *n* = 4; and OE/OE, *n* = 5; P7 WT, *n* = 5; OE/+, *n* = 5; and OE/OE, *n* = 3; and P21 WT, *n* = 5; OE/+, *n* = 4; and OE/OE, *n* = 4;. One-way ANOVA with Tukey's comparison: *p < 0.05, **p < 0.01, ***p < 0.001, ****p < 0.0001. ***I***, The number of axons in a 1:1 relationship and >1.5 μm in diameter that remain unmyelinated at P1, P7, and P21 in nerves of WT, c-Jun OE/+, and c-Jun OE/OE mice. Note the increase in unmyelinated axons in OE/OE nerves at P7 and P21, but at P7 only in OE/+ mice. P1 WT, *n* = 5; OE/+, *n* = 4; and OE/OE, *n* = 5; P7 WT, *n* = 5; OE/+, *n* = 5; and OE/OE, *n* = 3; and P21 WT, *n* = 5; OE/+, *n* = 4; and OE/OE, *n* = 4. One-way ANOVA with Tukey's comparison: **p < 0.01, ***p < 0.001, ****p < 0.0001. ***J***, Reduction in myelin thickness, measured as g-ratios, which is seen in the adults in both OE/+ and OE/OE nerves, is already present at P7 and P21. P7 WT, *n* = 5; OE/+, *n* = 5; and OE/OE, *n* = 3; and P21 WT, *n* = 5; OE/+, *n* = 4; and OE/OE, *n* = 4. The whiskers extend from the 5th to the 95th percentiles. One-way ANOVA with Tukey's comparison: *p < 0.05, **p < 0.01, ***p < 0.001. ***K***, The percentage of unmyelinated axons that are >1.5 μm in diameter in Schwann cell families or Remak bundles at P1, P7, and P21 in nerves of WT, c-Jun OE/+, and c-Jun OE/OE mice. Abnormally high numbers are seen in OE/OE nerves only. P1 WT, *n* = 5; OE/+, *n* = 4; and OE/OE, *n* = 5; P7 WT, *n* = 5; OE/+, *n* = 5; and OE/OE, *n* = 3; and P21 WT, *n* = 5; OE/+, *n* = 4; and OE/OE, *n* = 4. One-way ANOVA with Tukey's comparison: *p < 0.05, **p < 0.01, ***p < 0.001. ***L***, Western blot of nerve extracts from P7 WT (*n* = 3), OE/+ (*n* = 3), and OE/OE (*n* = 3) sciatic nerves showing cyclin D1, an indicator of cell proliferation. Quantification of the data are normalized to levels in P7 WT nerve, which are set as 1. Cyclin D1 levels are similar in all mouse lines. One-way ANOVA with Tukey's comparison: *p* = 0.3871. ***M***, Counts of Ki67-positive/Sox10-positive nuclei in P7 in WT (*n* = 6), OE/+ (*n* = 6), and OE/OE (*n* = 3) nerves. OE/OE nerves show increased Schwann cell proliferation. One-way ANOVA with Tukey's comparisons: *p = 00121. In ***E***–***K***, *p*-values are calculated relative to WT at the same age.

In developing nerves of c-Jun OE/+ mice, there was a trend toward c-Jun elevation at P1, but this was not significant, whereas at P7, Jun was elevated ∼6-fold compared with WT nerves at the same age. This failed to suppress levels of the myelin proteins Mpz and Krox20 in Western blots (Fig. 7*A*,*B*). However, nuclear Krox20 was reduced, as shown by double labeling of nerve sections with Krox20 and Sox10 antibodies to identify Schwann cells (Fig. 7*C*).

In developing nerves of c-Jun OE/OE mice, c-Jun levels at P7 were ∼8-fold that found in WT mice at that age and Mpz was suppressed in Western blots (Fig. 7*A*). Although Krox20 levels were not significantly reduced in Western blots, the number of Schwann cells that showed nuclear Krox20 was <50% of that in WT nerves, as seen in double immunolabeling of nerve sections (Figure 7*B*,*C*).

Electron microscopy at P1, P7, and P21 showed that, in c-Jun OE/+ mice, myelination was transiently delayed at P7, whereas in c-Jun OE/OE mice, myelination was severely inhibited (Fig. 7*D*).

In c-Jun OE/+ mice, nerve area and the percentage of >1.5-μm-diameter axons that were found within Schwann cell families or Remak bundles, both of which were normal in the adult, were also normal during development at all 3 time points (Fig. 7*E*,*K*). However, a number of other parameters were abnormal at P7, although they were normal at P1 and P21, revealing a transient delay in myelination. This includes the number of Schwann cell nuclei, which was elevated (Fig. 7*F*); the percentage of segregated (1:1), myelin-competent (>1.5 μm diameter) axons that were myelinated, which was reduced; the total number of myelinated axons, which was reduced (Fig. 7*H*); and the number of segregated (1:1) myelin-competent (>1.5 μm diameter) axons that were not myelinated, which was elevated (Fig. 7*I*). In adult nerves of these mice, the myelin sheaths were slightly thinner than in WT and this difference was already present at P7 and P21 (Fig. 7*J*).

In the developing c-Jun OE/OE nerves, nerve area was not significantly different from that seen in WT or c-Jun OE/+ mice (Fig. 7*E*). The large nerve area in P60 nerves of these mice therefore emerges in adulthood. At P7 and P21, these nerves contained ∼2× the number of Schwann cell nuclei seen in WT nerves, a smaller difference than that seen in the adult (Fig. 7*F*). This suggests ongoing, low-level Schwann cell proliferation in adult mutant nerves, supported by Ki67 labeling of Sox10-positive Schwann cells, although the differences between WT and c-Jun OE/OE nerves in cyclin D1 levels and did not reach significance (Fig. 7*L*,*M*). In other respects, the differences between developing WT and mutant nerves at P7 and P21 already matched those seen in adult P60 nerves. This includes a reduced number of myelinated axons, an increased number of segregated myelin competent axons that remained unmyelinated, thinner myelin sheaths, and an increased number of >1.5-μm-diameter axons that were seen within Schwann cell families or Remak bundles (Fig. 6*G–K*).

These experiments show that c-Jun negatively regulates developmental myelination in a dose-dependent manner. In the c-Jun OE/+ mouse, ∼6-fold overexpression of c-Jun results in a transient delay at P7, whereas the nerve had recovered at P21. Conversely, in the developing nerves of c-Jun OE/OE mice, in which c-Jun levels are ∼50% higher than in c-Jun OE/+ nerves, myelination is permanently inhibited and seen in only 30–40% of >1.5-μm-diameter myelin-competent axons, a figure comparable to that seen in the adult.

### Remyelination after nerve injury in c-Jun OE/+ mice

c-Jun is a key amplifier of the repair Schwann cell phenotype, which is generated in the distal stump of injured nerves. Therefore, elevation of c-Jun is a candidate approach for improving nerve repair under conditions where it falters, such as in older animals or due to long-term Schwann cell denervation (Wagstaff et al., 2017). The observation that, in c-Jun OE/+ mice, adult nerves with ∼6-fold elevation of c-Jun achieve a relatively normal degree of nerve architecture and myelination during development, albeit with a delay, is encouraging for this approach because it demonstrates that significant c-Jun elevation and myelination are compatible. However, after injury, remyelination is slower and more easily disrupted than developmental myelination. The two processes are also partly controlled by distinct signals. We therefore tested the capacity of c-Jun OE/+ nerves to remyelinate after nerve injury.

**Figure 8.**
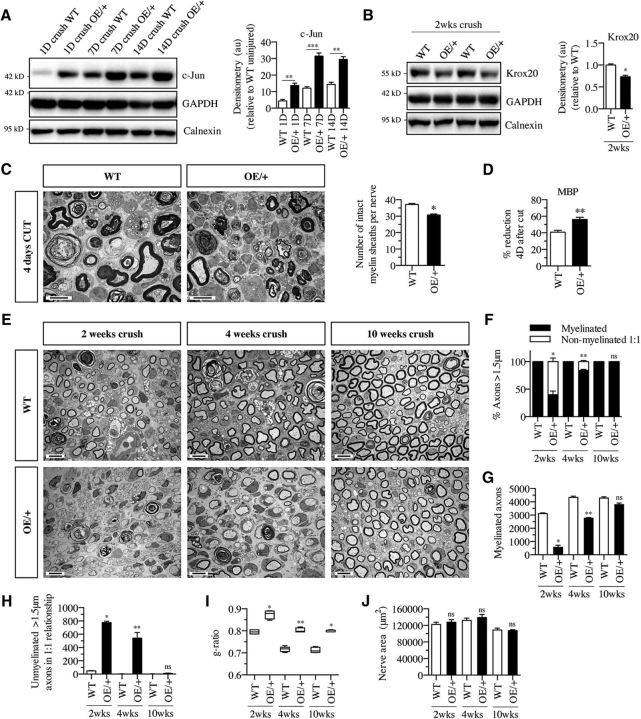
Remyelination of OE/+ nerves is delayed. ***A***, Western blot of c-Jun in nerve extracts from the distal stump of adult WT (*n* = 4) and OE/+ (*n* = 4) nerves 1 d, 7 d, and 2 weeks after crush. The graph shows quantification of the results normalized to levels in uninjured WT nerves, which are set as 1. Note significant elevation of c-Jun at all time points. Mann–Whitney *U* test: 1 d, *p* = 0.0006 (*n* = 4); 7 d, *p* = 0.0002 (*n* = 4); and 2 weeks, *p* = 0.0022 (*n* = 4). ***B***, Western blot of nerve extracts from the distal stump of adult WT and OE/+ nerves 2 weeks after crush. The results are quantified in the graph and normalized to levels in 2 week crushed WT nerve, which are set as 1. Krox20 levels are reduced in OE/+ nerves. Mann–Whitney *U* test: *p* = 0.0286 (*n* = 4). ***C***, Representative electron micrographs from the distal stump 4 d after sciatic nerve cut in WT and c-Jun OE/+ mice illustrating collapsed myelin sheaths. The graph shows that fewer intact myelin sheaths per nerve profile remain in OE/+ nerves than in WT. Mann–Whitney *U* test: *p* = 0.0286 (*n* = 4). ***D***, Transected c-Jun OE/+ nerves clear myelin protein faster than WT nerves. The graph shows the reduction in MBP 4 d after transection expressed as a percentage of MBP in uninjured nerve. WT and c-Jun OE/+ nerves have cleared close to 40% and 60% of their MBP content, respectively. The data are obtained from quantitation of Western blots. WT, *n* = 4; OE/+, *n* = 8. Mann–Whitney *U* test: *p* = 0.0070. ***E***, Representative electron micrographs from the distal stump of WT and OE/+ nerves 2, 4, and 10 weeks after crush. In OE/+ nerves, the number of myelinated axons, which is reduced at 2 and 4 weeks, has recovered at 10 weeks. Scale bar, 5 μm. ***F***, The percentage of axons >1.5 μm in diameter and in a 1:1 ratio that are myelinated in the distal stump of WT and c-Jun OE/+ mice 2, 4, and 10 weeks after nerve crush. Note that myelination in OE/+ nerves, which is reduced at 2 weeks, has recovered substantially by 4 weeks and is normal at 10 weeks. Mann–Whitney *U* test: 2 weeks, *p* = 0.0286 (*n* = 4); 4 weeks, *p* = 0.0079 (*n* = 5); and 10 weeks, *p* = 0.0571 (*n* = 4). ***G***, The number of myelinated axons per nerve profile of the distal stump of WT and c-Jun OE/+ mice 2, 4, and 10 weeks after nerve crush. In c-Jun OE/+ mice, few myelinated axons are present at 2 weeks, but normal numbers are seen at 10 weeks. Mann–Whitney *U* test: 2 weeks, *p* = 0.0286 (*n* = 4); 4 weeks, *p* = 0.0079 (*n* = 5); and 10 weeks, *p* = 0.0571 (*n* = 4). ***H***, The number of unmyelinated axons >1.5 μm in diameter and in a 1:1 relationship that have not myelinated in the distal stump of WT and OE/+ nerves 2, 4, and 10 weeks after crush. Two and 4 week crushed OE/+ nerves contain elevated numbers of umyelinated axons, but their number has fallen to normal levels at 10 weeks. Mann–Whitney *U* test: 2 weeks, *p* = 0.0286 (*n* = 4); 4 weeks, *p* = 0.0079 (*n* = 5); and 10 weeks, *p* = 0.1143 (*n* = 4). ***I***, Myelin thickness, measured as g-ratios, is reduced in the distal stump of OE/+ nerves at all time points after crush. The whiskers extend from the 5th to the 95th percentiles. Mann–Whitney *U* test: 2 weeks, *p* = 0.0286 (*n* = 4); 4 weeks, *p* = 0.0079 (*n* = 5); and 10 weeks, *p* = 0.0286 (*n* = 4). ***J***, The area of transverse sections through the distal stump 2, 4, and 10 weeks after crush is similar in WT and OE/+ nerves. Mann–Whitney *U* test: 2 weeks, *p* = 0.6571 (*n* = 4); 4 weeks, *p* = 0.3095 (*n* = 5); and 10 weeks, *p* = 0.9004 (*n* = 4). In ***A*** and ***F***–***J***, *p*-values are calculated relative to WT at the same time after injury.

After sciatic nerve crush injury, c-Jun levels distal to the crush were elevated in WT mice and this elevation was enhanced in c-Jun OE/+ mice, as expected (Fig. 8*A*). At 1, 7, and 14 d after injury, c-Jun levels in OE/+ nerves were 2- to 3-fold higher than those in crushed WT control nerves. This amounted to an ∼12-fold (at 1 d after crush) to an ∼30-fold (at 7 and 14 d after crush) elevation of c-Jun in crushed c-Jun OE/+ nerves compared with the levels found in uninjured control nerves. This was accompanied by somewhat lower Krox20 levels (Fig. 8*B*). At 21 d after crush, c-Jun levels in WT nerves had declined, although they remained significantly above those in uninjured nerves (data not shown).

When examined 4 d after nerve cut, nerves of c-Jun OE/+ mice showed accelerated collapse/breakdown of myelin sheaths and faster clearance of the myelin protein MBP, in agreement with previous evidence that c-Jun promotes myelin clearance and myelin autophagy (Arthur-Farraj et al., 2012; Gomez-Sanchez et al., 2015) (Fig. 8*C*,*D*).

Examination of crushed c-Jun OE/+ nerves by electron microscopy showed a significant delay in remyelination at 2 weeks after nerve crush (Fig. 8*E*). At this time point, ∼35% of myelin-competent (>1,5 μm) axons were myelinated in OE/+ nerves, whereas >95% were myelinated in WT nerves (Fig. 8*F*). At 2 weeks after crush, the number of myelinated axons was also reduced in OE/+ nerves (Fig. 8*G*) and the number of segregated, myelin-competent axons without myelin was elevated (Fig. 8*H*). Significant recovery was seen 4 weeks after crush, when ∼75% of myelin-competent axons were myelinated in OE/+ nerves compared with 98% in WT nerves. By 10 weeks, essentially all myelin-competent axons were myelinated in both genotypes, a situation similar to that in uninjured nerves of these mice (Fig. 8*E–H*). Myelin sheaths in adult c-Jun OE/+ mice are thinner than in WT (see previous section) and this difference was also seen in remyelinated nerves (Fig. 8*I*). Tumors were not seen and regenerating WT and c-Jun OE/+ nerves did not differ in size (Fig. 8*J*) or other aspects of general nerve architecture (Fig. 8*E*).

**Figure 9.**
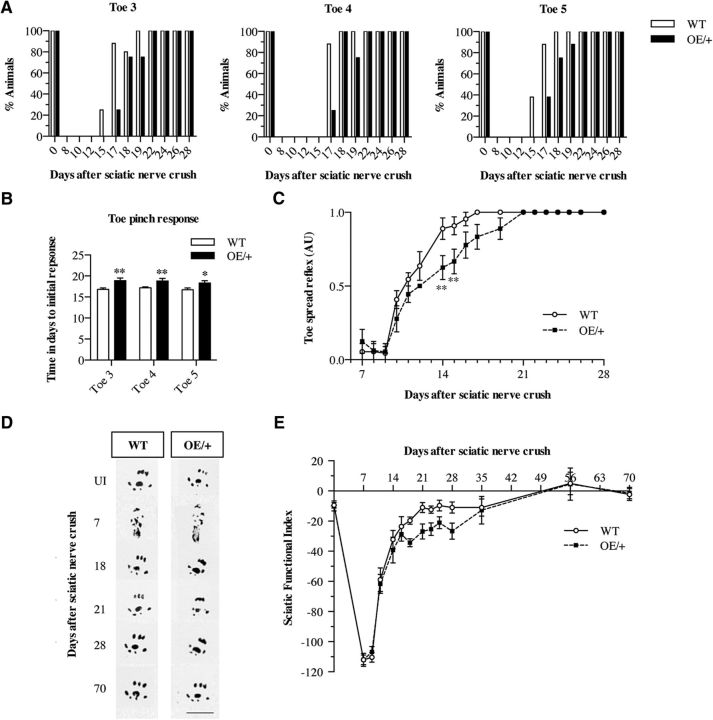
Functional recovery is slightly delayed in c-Jun OE/+ mice. ***A***, Toe pinch assay showing the percentage of mice that show a response to a pinch of toes 3, 4, and 5 at different times after sciatic nerve crush in WT and c-Jun OE/+ mice. In c-Jun OE/+ mice, all toes show a trend toward a delayed response. ***B***, The average time in days after crush at which the first toe pinch response is seen in toes 3, 4, and 5. WT, *n* = 10; OE/+, *n* = 9. Mann–Whitney *U* test: *p*-values are calculated relative to WT for each toe. Toe 3, *p* = 0.0056; toe 4, *p* = 0.0043; toe 5, *p* = 0.0404. ***C***, The toe spread reflex in WT (*n* = 10) and OE/+ (*n* = 9) mice after sciatic nerve crush. The reflex response is delayed at days 12, 14, and 15 in c-Jun OE/+ mice. Two-way ANOVA with Bonferroni's comparison: *p* = 0.0171. ***D***, Representative digital footprints from WT and c-Jun OE/+ mice taken at 0, 7, 18, 21, 28, and 70 d after sciatic nerve crush used in sciatic functional index (SFI) analysis. ***E***, SFI results from WT (*n* = 11) and c-Jun OE/+ (*n* = 8) mice at different times after sciatic nerve crush. There is no significant difference between WT and c-Jun OE/+ mice. Two-way ANOVA with Bonferroni's comparison: *p* = 0.5545.

Importantly, the c-Jun OE/+ mice achieved full functional recovery after nerve crush. In the toe pinch test, which is primarily a sensory test, time to full recovery was comparable in WT and c-Jun OE/+ mice, whereas time to initial response (group average) was ∼2 d longer in the mutants (Fig. 9*A*,*B*). In the toe spread reflex, primarily a test of motor recovery, c-Jun OE/+ mice showed a transient delay in recovery on days 14 and 15 only (Fig. 9*C*), possibly caused by delay in myelination at this time point (Fig. 8*F*,*G*). In the sciatic functional index, a sensory–motor test, c-Jun OE/+ mice showed a nonsignificant trend toward a transient delay during the second and third week after injury (Fig. 9*D*,*E*).

## Discussion

We have generated c-Jun OE/+ and c-Jun OE/OE mice with forced expression of c-Jun in Schwann cell nuclei to study the effects of a graded increase in c-Jun expression on Schwann cell development and on remyelination after injury. First, we have shown that, during development and in adult nerves, Schwann cells are remarkably tolerant of elevated c-Jun levels. Although developing and adult nerves of c-Jun OE/+ mice showed an ∼6-fold increase in c-Jun relative to WT nerves at the same age, myelination is only transiently affected at P7. By P21, myelination appears normal and, in the adult, Schwann cells and nerve architecture are similar to that in WT nerves, with the exception of modestly reduced myelin thickness, which is unlikely to have significant consequences for sensory–motor control. Second, although remyelination after injury, which generally is more easily perturbed than in development, is delayed in c-Jun OE/+ mice, remyelination shows strong recovery at 4 weeks and essentially all myelin-competent axons are myelinated at 10 weeks after injury. As in uninjured nerves, the myelin sheaths of regenerated OE/+ nerves remain thinner than those in regenerated WT nerves. The sensory and motor tests used here show only a slight delay followed by complete functional recovery in c-Jun OE/+ mice. Therefore, the c-Jun elevation in c-Jun OE/+ mice is compatible with essentially normal restoration of myelin and nerve function similar to that found before injury. This is important because we find that the c-Jun elevation in c-Jun OE/+ mice is sufficient to accelerate regeneration under conditions in which it is compromised by aging or long-term denervation (Wagstaff et al., 2017; L.J. Wagstaff, J. Gomez-Sanchez, R. Mirsky, and K.R. Jessen, unpublished data). Third, the higher overexpression achieved in c-Jun OE/OE mice confirms the potential of c-Jun to negatively regulate myelination, as seen previously *in vitro*. Myelination is strongly impaired during development and this persists in adult nerves, which show hypomyelinating pathology, enlarged connective tissue, and immature onion bulbs. Fourth, even in nerves of aged c-Jun OE/OE mice, there is no evidence of tumor formation and these nerves show strong activation of the tumor suppressor P19^ARF^. The absence of tumorigenic effect of enforced c-Jun expression in Schwann cells is in agreement with the fact that mechanical nerve damage is not associated with tumor formation, although injured WT nerves contain proliferating cells with high c-Jun levels.

c-Jun is involved directly or indirectly in the control of∼180 of the ∼4000 genes that change significantly after nerve injury. This allows c-Jun to take part in the regulation of a spectrum of properties of denervated repair Schwann cells, including morphology, autophagy-mediated myelin breakdown, and the expression of trophic factors linked to regeneration, including GDNF, artemin, BDNF, NGF, and LIF (Eggers et al. 2010; Arthur-Farraj et al., 2012; Fontana et al., 2012; Gomez-Sanchez et al., 2015, Jessen and Mirsky, 2016; Jessen et al., 2015a). Of these, GDNF, artemin, and LIF have been shown to be direct targets of c-Jun. Additional evidence for direct regulation of injury-induced genes by c-Jun comes from a study of enhancer activation in Schwann cells. This showed c-Jun-binding sites associated with injury-activated enhancers of genes elevated after nerve injury, including Shh, Olig1, and Runx2 (Hung et al., 2015).

The gene targets and function of AP-1 transcription factors, a family to which c-Jun belongs, are regulated by dimerization partners and ancillary proteins (Chinenov and Kerrpola, 2001; Eferl and Wagner, 2003). Little is known about these components in Schwann cells.

RNA sequencing analysis showed that, in uninjured nerves of c-Jun OE/+ mice, 95 genes were expressed at levels that differed ≥2-fold from those in WT nerves. Sixty-seven of these genes were upregulated in response to increased c-Jun levels including *Shh.* In c-Jun OE/OE nerves, 1964 genes were changed ≥2-fold and 909 of these were upregulated, among them *GDNF*, *Shh*, *Olig1*, *Id2*, *Sox2*, and *Runx2.* The myelin genes *Mpz*, *Mbp*, and *Pmp22* were all strongly downregulated in c-Jun OE/OE nerves. Examining injured nerves, we previously identified 172 genes that were expressed at different levels in 7 d cut nerves of mice, in which c-Jun was genetically inactivated compared with inured WT nerves. Of these 172 genes, 106 were upregulated by higher c-Jun levels, namely expressed more highly in cut WT nerves than in cut c-Jun knock-out nerves. A comparison of the 15 genes most upregulated by c-Jun in uninjured c-Jun OE/+ and OE/OE nerves in the present work with the 15 genes that are most upregulated by c-Jun in 7 d cut nerves reveals only two common genes, *Shh* and *GDNF*. This limited similarity indicates that the group of genes directly or indirectly regulated by c-Jun in Schwann cells that have adopted the repair phenotype after injury is significantly different from the set of genes, which responds to c-Jun in cells that ensheath axons, many of which retain myelin differentiation.

We find that the substantial c-Jun elevation in c-Jun OE/OE mice is sufficient to cause severe hypomyelination. This is undoubtedly related to the ability of c-Jun to suppress myelin genes. Although the causal relationship between the increased c-Jun levels seen in human CMT1A and demyelination has not been analyzed (Hutton et al., 2011), it seems clear that sustained dys-regulation of c-Jun resulting in high expression in uninjured nerves is a potential hazard. c-Jun is therefore a candidate for a factor that could cause or promote pathological demyelination.

c-Jun OE/OE nerves and nerves affected by demyelinating neuropathies, in particular CMT1A, show many similarities, most obviously hypomyelination. In c-Jun OE /OE mice, this involves substantially thinner myelin sheaths and an ∼40% reduction in myelination among axons that have segregated and are myelin competent. Axonal sorting is also adversely affected in younger (P60) c-Jun OE/OE mice because, in these mice, the percentage of unsorted >1.5-μm-diameter axons that remain in Remak bundles is 28% compared with <1% in WT. In common with human CMT1A nerves and nerves of the C22 and My41 mouse models of CMT1A, c-Jun OE/OE nerves also contain increased Schwann cell numbers (Robertson et al., 2002; Lupski and Chance, 2005). However, neither mouse models of CMT1A nor the c-Jun OE/OE mice show a significant numbers of Schwann cells that are without axonal contact. Increase in endoneurial connective tissue, which is substantial in c-Jun OE/OE mice, is also a feature of human CMT1A nerves and of the CMT1A rat (Palumbo et al., 2002; Lupski and Chance, 2005; Fledrich et al., 2012). Abnormalities of Remak fibers, including the formation of membranous structures that do not contact axons, which are seen in c-Jun OE/OE mice, are also described in the My41, C22, and C3 mouse models of CMT1A (Robertson et al., 2002; Verhamme et al., 2011). Last, onion bulbs, which are prominent in human CMT1A nerves and seen in rodent CMT1A models (Lupski and Chance, 2005; Fledrich et al., 2012), are also present in nerves of aged c-Jun OE/OE mice.

The histological changes outlined here for c-Jun OE/OE and CMT1A nerves are generally not specific to these conditions, but are also observed to a varying degree in a number of other non-tumor-associated human nerve pathologies, mutant mouse nerves, or in injured nerves. (Low, 1977; Haney et al., 1999; Chen et al., 2003; Ling et al., 2005; Kagitani-Shimono et al., 2008; Ishii et al., 2016). The relative paucity of disease-specific structural changes in pathological nerves and the sloppy relationship between molecular and histological phenotype makes it hard to interpret a particular histology in terms of a causal sequence.

Although the availability of binding partners or other ancillary proteins are important regulators of c-Jun function, the levels of c-Jun protein are likely to be a key factor in determining whether c-Jun has beneficial or adverse effects on nerve biology. Previous work shows that, already at low or moderate levels compatible with myelination, c-Jun appears to promote neuron-supportive signaling from Schwann cells to neurons, including the activation of trophic factors such as GDNF, whereas higher levels are required to suppress myelin genes. Therefore, in the C3 mouse model of CMT1A, c-Jun is elevated, but this is not high enough to disrupt myelin, although it enhances axonal survival and sensory motor performance (Hantke et al., 2014). Similarly, in CMT1X mice, c-Jun is elevated and increases GDNF expression, but does not disrupt myelination (Klein et al., 2014).

In sum, the present results show that, although moderate c-Jun increase is well tolerated during Schwann cell development and remyelination after injury, strong elevation of c-Jun in uninjured nerves suffices to induce significant hypomyelination pathology, implicating c-Jun in demyelinating neuropathies. Conversely, we did not find a link between c-Jun elevation and tumorigenesis, which is consistent with the fact that tumors do not form after nerve injury, although c-Jun is strikingly elevated as Schwann cells lose myelin differentiation and proliferate. We also found that, after crush injury of OE/+ nerves, myelination and nerve function can be restored in the presence of c-Jun levels that are high enough to promote axonal regeneration in mice in which regeneration has been compromised by long-term denervation or advanced age.
